# Efficacy and cost-effectiveness of a therapist-assisted web-based intervention for depression and anxiety in patients with ischemic heart disease attending cardiac rehabilitation [eMindYourHeart trial]: a randomised controlled trial protocol

**DOI:** 10.1186/s12872-020-01801-w

**Published:** 2021-01-07

**Authors:** Susanne S. Pedersen, Christina M. Andersen, Robert Ahm, Søren J. Skovbakke, Robin Kok, Charlotte Helmark, Uffe K. Wiil, Thomas Schmidt, Kim Rose Olsen, Jacob Hjelmborg, Ann-Dorthe Zwisler, Lisbeth Frostholm

**Affiliations:** 1 Department of Psychology, University of Southern Denmark, Campusvej 55, DK-5230 Odense M, Denmark; 2 Department of Cardiology, Odense University Hospital, Odense, Denmark; 3 Open Patient data Explorative Network (OPEN), Odense University Hospital, Odense, Denmark; 4 Department of Cardiology, Zealand University Hospital, Roskilde, Denmark; 5 The Maersk Mc-Kinney Moller Institute, University of Southern Denmark, Odense, Denmark; 6 Danish Center for Health Economics (DaCHE), Odense University Hospital and University of Southern Denmark, Odense, Denmark; 7 Epidemiology, Biostatistics and Biodemography, University of Southern Denmark, Odense, Denmark; 8 Danish Knowledge Center for Rehabilitation and Palliative Care (REHPA), Odense University Hospital and University of Southern Denmark, Odense, Denmark; 9 Research Clinic for Functional Disorders and Psychosomatics, Aarhus University Hospital, Aarhus, Denmark

**Keywords:** Acceptance and commitment therapy, Anxiety, Cardiac rehabilitation, Cognitive behavioral therapy, Depression, Dropout, eHealth intervention, Heart disease, Quality of life

## Abstract

**Background:**

One in five patients with ischaemic heart disease (IHD) develop comorbid depression or anxiety. Depression is associated with risk of non-adherence to cardiac rehabilitation (CR) and dropout, inadequate risk factor management, poor quality of life (QoL), increased healthcare costs and premature death. In 2020, IHD and depression are expected to be among the top contributors to the disease-burden worldwide. Hence, it is paramount to treat both the underlying somatic disease as well as depression and anxiety. *eMindYourHeart* will evaluate the efficacy and cost-effectiveness of a therapist-assisted eHealth intervention targeting depression and anxiety in patients with IHD, which may help fill this gap in clinical care.

**Methods:**

*eMindYourHeart* is a multi-center, two-armed, unblinded randomised controlled trial that will compare a therapist-assisted eHealth intervention to treatment as usual in 188 CR patients with IHD and comorbid depression or anxiety. The primary outcome of the trial is symptoms of depression, measured with the Hospital Anxiety and Depression Scale (HADS) at 3 months. Secondary outcomes evaluated at 3, 6, and 12 months include symptoms of depression and anxiety (HADS), perceived stress, health complaints, QoL (HeartQoL), trial dropout (number of patients dropped out in either arm at 3 months) and cost-effectiveness.

**Discussion:**

To our knowledge, this is the first trial to evaluate both the efficacy and cost-effectiveness of a therapist-assisted eHealth intervention in patients with IHD and comorbid psychological distress as part of CR. Integrating screening for and treatment of depression and anxiety into standard CR may decrease dropout and facilitate better risk factor management, as it is presented as “one package” to patients, and they can access the *eMindYourHeart* program in their own time and at their own convenience. The trial holds a strong potential for improving the quality of care for an increasing population of patients with IHD and comorbid depression, anxiety or both, with likely benefits to patients, families, and society at large due to potential reductions in direct and indirect costs, if proven successful.

*Trial registration* The trial was prospectively registered on https://clinicaltrials.gov/ct2/show/NCT04172974 on November 21, 2019 with registration number [NCT04172974].

## Background

Ischaemic heart disease (IHD) is a chronic disease, characterised by reduced blood supply to the heart due to the build-up of plaque in the coronary arteries that may lead to a heart attack – an acute myocardial infarction (AMI). The number of patients living with IHD has increased exponentially due to ageing of the population and more patients surviving due to better treatment options [1]. In Denmark alone, 21,000 new patients are diagnosed with IHD every year [2].

An AMI often comes out of the blue and comprises a threat to the patient’s life and sense of self. The sudden confrontation with a life-threatening illness may trigger different psychological responses. Not surprisingly, 20% of patients suffer from depression, anxiety or both [3]. Both a clinical diagnosis and subthreshold levels – often undetected – comprise barriers for lifestyle changes, increase risk of non-adherence, refusal *or* dropout from cardiac rehabilitation (CR), hospitalisation, and premature death [4–6]. The health economic impact associated with both IHD [7] and depression [8, 9] are high. Depression alone adds an extra cost of 33% compared to patients without depression [10]. Compounding the issue, both IHD and depression are among the top contributors to the disease-burden worldwide in 2020 [2, 11, 12], warranting that we treat both the underlying somatic disease and psychological comorbidity [13].

In order to mitigate the impact of IHD, national guidelines on CR have been implemented across the world. CR is a multi-disciplinary effort targeted to reduce progression of heart disease. The focus is on improving the patient’s functional level physically, mentally and socially, reducing activity-related symptoms, minimising disability, and improving QoL [14, 15]. There may be variability in programs. Evidence for an effect of CR is strong with significant reduction in mortality on top of state-of-the-art treatments, such as percutaneous coronary intervention and pharmacotherapy [16]. However, to obtain these benefits, it is paramount that patients are offered CR, consent to participate, and stay in the program. So far there is still room for improvement with respect to retention [17]. Numerous barriers for participation and adherence exist and include person-related factors (e.g. gender, socio-economic status, health literacy, and comorbidities), factors related to the CR program (e.g. accessibility, proximity to the CR setting) [18], and anxiety and depression [5, 18].

In 2013, the Danish Health Authority included screening for symptoms of depression and anxiety with the Hospital Anxiety and Depression Scale (HADS) in the national clinical guidelines on CR [19]. However, the CR setting has no treatment to offer patients who screen positive, except referring them to their general practitioner (GP). The Danish situation is not unique, as screening combined with treatment of patients’ distress is far from universally implemented [20].

### eHealth and its potential in the cardiac rehabilitation setting

*eMindYourHeart* represents an innovative solution to bridge this gap in clinical care [17], as it introduces a tailored therapist-assisted eHealth intervention targeting depression and anxiety that is added to standard CR. Internet-delivered treatment for common mental disorders has been investigated in well over 100 randomised controlled trials (RCTs) and are as effective as face-to-face-treatment [21–24]. Advantages of internet-delivered treatment include that patients can access the intervention anytime from anywhere, work at their own pace, and avoid taking time off work to see a psychologist. This may reduce barriers for engaging in treatment as part of CR [25] and societal costs, since patients do not need to take time off work. Due to the flexibility of the mode, it may also be more easy for patients to integrate treatment into their daily lives [26]. An online treatment approach also secures continuity of treatment for patients even during pandemics like COVID-19, when hospitals largely close down for patients with less acute needs.

Only few studies [27–29] have examined the efficacy of an approach like *eMindYourHeart* to treat compromised mental health in patients with IHD, with the studies showing mixed results. Two studies delivered such intervention in addition to traditional CR [30]. To our knowledge, no studies have examined both the efficacy and cost-effectiveness of such interventions in patients with IHD referred to CR. High quality studies are warranted due to the current moderate quality level of trials in this field [30]. These programs should not be one-size-fits-all but targeted to the individual patient’s needs and preferences [17].

### Objectives and hypotheses

#### Primary objective

The primary objective is to evaluate the efficacy of the eHealth intervention *eMindYourHeart* targeting depression and anxiety + treatment as usual (TAU) (i.e. current CR) as compared to TAU alone on symptoms of depression at 3 months (end of the intervention). We chose TAU as the comparator, as we want to examine whether our intervention improves patients’ depression and anxiety outcomes. We chose depression as the primary outcome, as evidence on depression as a risk factor for mortality is more solid than evidence on anxiety [31].

#### Secondary objectives

The secondary objectives are to evaluate the efficacy of the eHealth intervention targeting depression and anxiety + TAU as compared to TAU alone on symptoms of anxiety, perceived stress, health complaints, QoL, and dropout (number of patients dropped out in either arms at 3 months) at 3 months, and symptoms of depression, anxiety, perceived stress, health complaints, and QoL at 6 and 12 months’ follow-up and cost-effectiveness/cost-utility.

#### Hypotheses

Given the user-centered, therapist-assisted eHealth approach to treat depression and anxiety, allowing patients to engage in the intervention when and where they want, we hypothesise that the intervention will be broadly acceptable to patients, have a low dropout rate and be effective and cost-effective.

## Methods

### Trial design

**Fig. 1:**
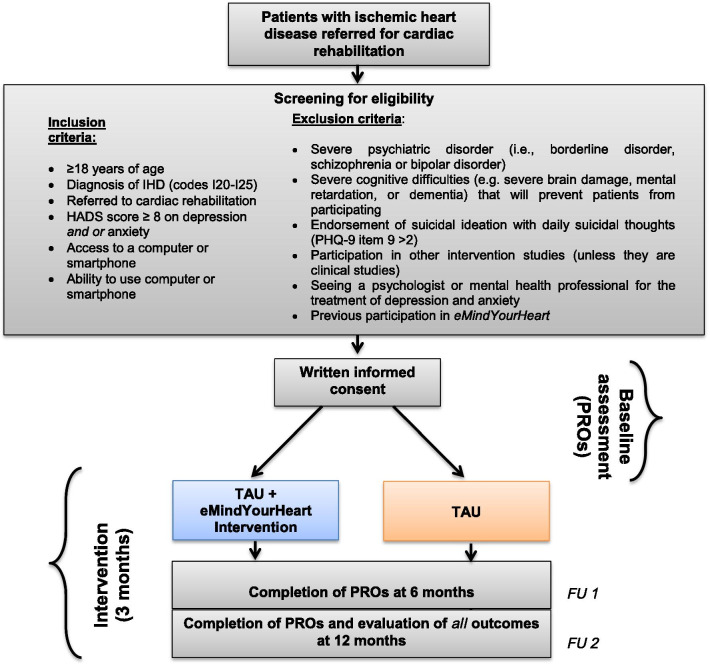
Trial design and flowchart of patient inclusion and follow-up

*eMindYourHeart* is a multi-center, two-armed, unblinded, parallel group RCT (see Fig. 1**)**. The trial will enroll patients from the CR settings at hospitals and municipalities delivering CR across all five regions in Denmark. We will use the TIDieR (Template for Intervention Description and Replication) guidelines and the CONSORT eHealth Checklist when reporting the results of the *eMindYourHeart* RCT [32, 33].

### Eligibility and recruitment

Consecutive patients referred to CR with IHD, ≥18 years of age, with a diagnosis of IHD (codes I20-I25), HADS score ≥ 8 on depression, anxiety or both, with access to a computer or smartphone, ability to use a computer or smartphone, and proficient in the Danish language are eligible for inclusion. Patients with severe psychiatric disorders (i.e., borderline disorder, schizophrenia or bipolar disorder), severe cognitive difficulties (e.g. severe brain damage, mental retardation, or dementia) that will prevent patients from participating, endorsement of suicidal ideation with daily suicidal thoughts (PHQ-9 item 9 > 2), participating in other intervention studies (unless they are clinical studies [e.g. medication trials]), or seeing a psychologist or mental health professional for the treatment of depression and anxiety, will be excluded (Fig. 1). Patients who were previously enrolled in the study and referred to CR due to a new cardiac event will not be eligible to participate a second time. Table 1 presents an overview of the recruitment procedure.

**Table 1:** Overview of recruitment procedure

• Patients will be recruited consecutively from the participating CR centres. • When the patient first visits the CR centre, one of the members of staff will screen the patient for symptoms of depression and anxiety. • If the patient has a positive score on depression and/or anxiety and complies with the additional inclusion criteria and none of the exclusion criteria, CR staff will inform the patient orally and in writing about the study. • Two scenarios are then possible: 1. If the patient is interested in participating in the study and does not wish additional time to contemplate the decision or to have a significant other present, informed consent will be signed by both parties. 2. If the patient is interested in participating in the trial and wishes either 24 h to contemplate the decision and/or to have a significant other present, they receive a link to a declaration of consent via e-boks. • After having signed the informed consent (the patient will receive a copy) the patient will be included into the study. • A note will be added to the patient’s electronic health records, if the patient is included into the study. • A link to the questionnaire to complete at baseline will be sent to the patient via e-boks. • When the therapist has received a confirmation that the questionnaire has been completed, and if the HADS score is still positive, the patient will be contacted by telephone by the therapist and take part in a short purpose-designed diagnostic interview. • Within 2 days, eligible patients are randomised to either TAU *or* TAU + the *eMindYourHeart* intervention by administrative staff from SDU and informed of the result by the therapist. • Patients allocated to TAU + the *eMindYourHeart* intervention will receive instructions on how to use/access the platform by the therapist. • Patients who do not wish to participate in the study are asked about *(i)* the reason and *(ii)* whether we may use the patient’s data from the rehabilitation settings and DHDR.	

## Sample size calculation

Based on Stauber et al. [34], a conservative estimate of the standard deviation of the mean of the HADS depression score at 3 months is 3.5. The minimal clinically important difference for the HADS is 2 points [35]. For a normal-based comparison of independent means, with a power of 90% (ß = 0.1) and a risk of Type 1 error of 5% (α = 0.05), the estimated required number of patients is *n* = 66 in each group. Considering a potential 30% dropout rate, the estimated number of needed participants is *n* = 94 patients in each group (n (total) = 188).

## Ethics and safety considerations

Ethical approval for the trial is obtained from the Regional Committees on Health Research Ethics for Southern Denmark [S-20180024] (version 5 approved 10 September 2019). The required permissions are obtained from the Danish Data Protection Agency via Odense University Hospital (17/41433 on November 24, 2017). The trial will be conducted according to the Helsinki Declaration and was prospectively registered on http://www.clinicaltrials.gov [NCT04172974]. All patients will receive oral and written information about the trial and sign an informed consent form. Patients who do not wish to participate in the trial are asked about *(i)* the reason and *(ii)* whether we may use the patient’s data from the rehabilitation setting and the Danish Cardiac Rehabilitation Database (DHRD). DHRD is a Danish quality database that collates health data on CR from all hospitals in Denmark to ensure cohesiveness and quality in CR in Denmark [36]. It is compulsory for all hospitals offering CR to report into the database.

Subjects can leave the trial at any time for any reason if they wish without any consequences. The investigator can decide to withdraw a subject from the trial due to urgent medical reasons. Subjects withdrawn from the trial will not be replaced and will not be followed-up. Subjects who drop out of the trial will be followed up according to the intention-to-treat principle [37].

There are no expected medical risks associated with trial participation, as medical treatment is provided according to national clinical guidelines [14] and the trial compares two types of care (i.e., *(i)* TAU + eHealth intervention versus *(ii)* TAU. However, we cannot rule out that the eHealth intervention may induce additional depression and anxiety in some patients [38]. The Ethics Committee does not require us to have a Data Safety Monitoring Board (DSMB), but annually we are required to report serious side effects and incidents to the Committee.

**Fig. 2:**
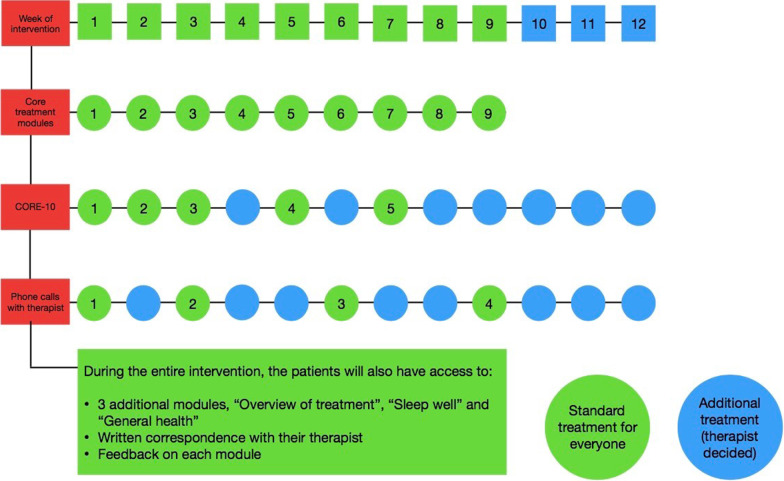
*eMindYourHeart* intervention and timeline

The treatment manual developed for the trial includes guidelines for therapists how to handle patients scoring high on suicidal ideation. Patients with a Patient Health Questionnaire (PHQ-9 item 9) > 2 will be excluded from the trial and will be asked to see their general practitioner (GP) and can be referred to *Livslinien* (Danish suicidal prevention hotline). All other patients will be included. Therapists also administer the CORE-10 to patients several times during the intervention to evaluate progress (Fig. 2). In case of deterioration, a contingency plan as written into the treatment protocol will be implemented that includes e.g. more frequent contact, more tailoring to meet patients’ needs, suicidal risk assessment, referral to GP for evaluation of psychopharmaca etc.

## Randomisation, blinding and treatment allocation

Patients will be randomised 1:1 to **(i)** TAU + *eMindYourHeart* intervention to treat psychological comorbidity versus **(ii)** TAU alone. The randomisation will be stratified by site and stable versus unstable IHD. Randomisation will be performed in the internationally recognised data capture platform – Research Electronic Data Capture (RedCap) – by an independent researcher who will be blinded to the allocation list. Patients and therapists will be informed of treatment allocation after randomisation is final. A merged blocks permutation stratified by inclusion centre will be used. This adaptation of a stratified block randomisation is better suited to small clinical trials where regular block randomisation would compromise predictability [39]. The randomisation list will be created using the mergedblocksmulti function of the mergedblocks package in R3.6.0 for Windows [40] and will only be accessible to the researcher who created the list (RK). Apart from the psychologists delivering the therapy, the research team will be blinded to treatment allocation. Due to the nature of the intervention, it is not possible to blind patients to their trial condition.

## Intervention

### Development

We developed the *eMindYourHeart* intervention specifically for and with involvement of the target group (patients with IHD and comorbid depression, anxiety or both), using a user-centred design.

### Contents

The *eMindYourHeart* intervention is based on the principles of Cognitive Behavioural Therapy and include aspects from Acceptance and Commitment Therapy and Compassion Focused Therapy [41]. The intervention consists of an introductory module, 9 core treatment modules and 2 optional modules (related to sleep and lifestyle changes, respectively) (Table 2). As part of the intervention, patients will be asked to complete the CORE-10 in weeks 1, 2, 3, 5 and 7 and in additional weeks if the psychologist considers it necessary. The CORE-10 is used as an evaluation tool in therapy to evaluate the patient’s progress (http://www.coreims.co.uk/About_Measurement_CORE_Tools.html). See Fig. 2 for the *eMindYourHeart* intervention and timeline. Patients will also receive 4 phone calls as part of the intervention in weeks 1, 3, 6, and 9 (Fig. 2**)** and on a need to basis, as determined by the psychologist.

**Table 2:**
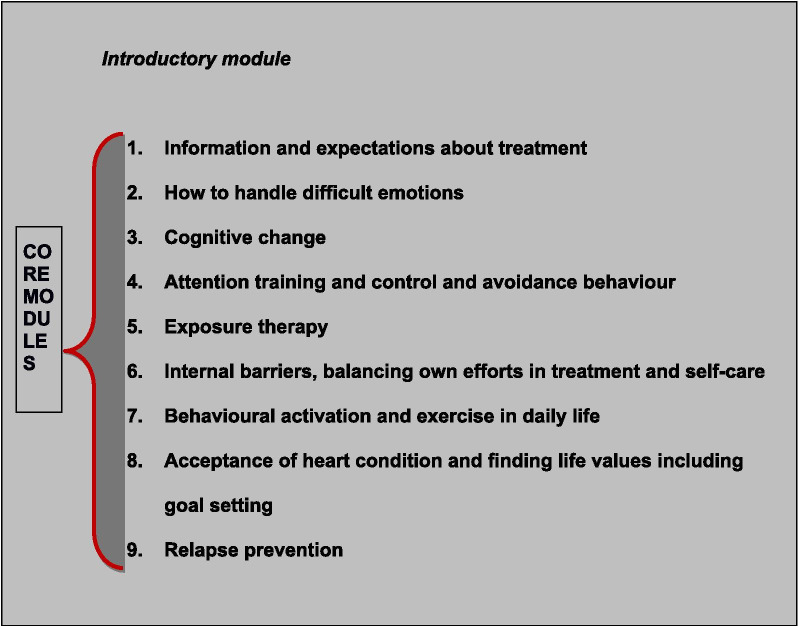
Overview of the modules of the *eMindYourHeart* intervention

Psychologists will interview patients randomised to the intervention arm over the phone, using motivational interviewing and a brief purpose-designed interview protocol that includes elements from the Schedules for Clinical Assessment in Neuropsychiatry (SCAN) interview. The purpose of the interview is to gain information about the patient and the patient’s situation, equivalent to a standard anamnestic assessment.

Patients are expected to complete the intervention within a 12-week period. All patients will have access to the intervention up to 6 months post intervention, enabling patients to revisit the material and reuse it ad libitum. The intervention is protocolised by means of a treatment manual to ensure fidelity and is somewhat flexibly targetable to the individual patient’s needs and preferences. These include adding additional time to the treatment from the standard 9 weeks all the way to 12 weeks, helping patients with sleep and lifestyle changes through the extra (optional) modules, giving specific psychological tools based on CBT to patients with needs not addressed in the standard treatment [e.g. stress management tools], and the possibility of extra phone calls.

Patients will gradually work through the modules and communicate with the psychologists via a secure online platform through asynchronous text messages. The therapist receives messages from the patient and notifications about the patient’s treatment course. Psychologists and master’s students in psychology from the University of Southern Denmark will provide the intervention. They are supervised by licensed psychologists, who are part of the project team. Internet-delivered treatment targets the same core treatment processes as in face-to-face treatment – only the mode of delivery is different [23].

### Delivery

Patients in the intervention group will receive access to a General Data Protection Regulation (GDPR) Directive compliant platform. Users login with their personal digital signature from the public Danish authorities (NemID). The eHealth platform/app is built around the core of the Drupal Content Management System and written in the programming language PHP. The Drupal platform is designed and maintained by a global community of thousands of programmers. Drupal is available under the GNU General Public License v2, which allows for free use, modification and distribution.

## Treatment as usual

All patients, irrespective of treatment condition that they are assigned to, will receive TAU. In Denmark, patients with IHD are referred to CR 1–2 weeks after discharge from the hospital, with CR focusing on physical training, risk factor control and clinical follow-up and maintenance of the patients’ targets, patient education (e.g. nutrition, smoking etc.), screening for depression and anxiety, and addressing psychosocial issues [14, 15].

## Trial outcomes

Primary: Symptoms of depression, measured with HADS at the end of the intervention (i.e., 3 months).

Secondary: Symptoms of anxiety measured with HADS at 3 months; symptoms of depression and anxiety at 6- and 12 months’ follow-up; QoL (HeartQoL) at 3, 6, and 12 months follow-up; trial dropout (number of patients dropped out in either arms at 3 months) and cost-effectiveness/cost-utility.

## Patient-reported measures

Table 3 provides an overview of the patient-reported and related measures and their time of assessment used in *eMindYourHeart.* Standardised and validated questionnaires are supplemented with purpose-designed questions (e.g. to tap into patient satisfaction with CR).

**Table 3:** Patient-reported outcome measures used and times of assessment

Construct assessed	Measure	Items	***Baseline***	***End of treatment***	***FU*** ***6 months***	***FU*** ***12 months***
Anxiety	Hospital Anxiety and Depression Scale (HADS-A)	7	X	X	X	X
Depressive symptoms	Hospital Anxiety and Depression Scale (HADS-D)	7	X	X	X	X
Anxiety symptoms	Cardiac Anxiety Questionnaire (CAQ-18)^1^	18	X	X	X	X
Depressive symptoms	Patient Health Questionnaire (PHQ-9)^1^	1	X	X	X	X
Loneliness	UCLA Loneliness Scale	3	X	X		
Social support	Multidimensional Scale of Perceived Social Support (MSPSS)	12	X			
Illness perceptions	Brief Illness Perceptions Questionnaire (B-IPQ)	9	X			
Quality of Life	HeartQoL	14	X	X	X	X
Perceived stress	Perceived Stress Scale	10	X	X	X	X
Health Anxiety and Somatic Complaints	Health Complaints Scale	24	X	X	X	X
Physical activity	Purpose-designed	2	X	X	X	X
Patient’s engagement in general CR	Purpose-designed	4		X		
Potentially negative effects of treatment	The Negative Effects Questionnaire (NEQ)^a^	20		X		
Cost-effectiveness/cost-utility^2^	EQ-5D-5L	6	X	X	X	X
Patient expectations to CR	The credibility/ expectancy questionnaire (CET)^a^	4	X			
Patient satisfaction with / evaluation of eHealth intervention	Internet evaluation and usability questionnaire^a^	6		X		
Patient satisfaction with CR	Purpose-designed measure	1		X		

*FU* Follow-up

^a^Questionnaire is only completed by patients in the intervention group [the CET is completed on the platform]

## Definition of treatment adherence and dropout

In internet-based therapy, research it is often unclear how treatment adherence is defined and there seems to be no gold standard [42]. However, use of different definitions across studies makes comparisons difficult with respect to efficacy of internet-based therapy. In *eMindYourHeart*, we define treatment adherence by the number of modules that the patient completes. Since treatment needs of individual patients may vary and we have elective modules that will be allocated to patients based on their needs, a module is considered completed, when the therapist judges that the individual patient has worked sufficiently with the exercises in the module. For example, if a patient primarily suffers from depression and not anxiety, the module focusing on exposure therapy is likely not relevant. The therapist’s judgement is made according to guidelines specified in the treatment manual. This ensures consistency across therapists and enhances the fidelity of the study.

A patient is considered a dropout, if the patient actively informs the therapist that the patient wishes to stop treatment. A patient who, at some point during treatment, stops adhering to treatment and does not respond to the therapist, will not be considered a dropout, but will stay enrolled in the treatment until the end of the 12-week treatment duration.

## Other measures

Information on patients’ demographic and clinical characteristics at baseline will be captured from patients’ medical records at the rehabilitation centre *or* the hospital, DHRD*, or* via purpose-designed questions. These questions will be included in the questionnaire sent to patients at baseline with standardised and validated measures (Table 3).

Measures of total health care costs measured in EUR will be collected from the Danish administrative health care registers. Loss of labour market productivity will be calculated using the human capital approach where average wages are used to estimate loss of work productivity. Costs will be measured in 2020 prices using a currency rate of 1 EURO to 7.45 DKK. Health care costs include all hospital utilisation aggregated using the Danish diagnosis-related group (DRG) prices (somatic and psychiatric care). Primary care services will be aggregated using national health insurance service fees (including GPs and private practicing specialists). Total utilisation of prescription drugs will be measured by purchasing prices of the patient excluding value added tax (VAT).

Cost-utility will be measured by estimation of the incremental costs for a gain in QoL. Costs will be covering intervention costs and 12-month total health care utilisation. Thus, the cost-utility analysis will be carried out using the perspective of the health care sector. QoL will be measured using HeartQoL at 3, 6 and 12 months. A probabilistic approach will be used to assess uncertainty and acceptability curves will estimated.

## Statistical methods

Data will be analysed according to the modified intention-to-treat principle (i.e., all patients with a 3-month evaluation will be analysed as randomised). The intention-to-treat principle will be applied from the time that patients have initiated treatment (i.e., after the brief interview protocol that includes elements from the SCAN interview and motivational interviewing). Categorical variables will be summarised as n (%), continuous variables as min-max, mean (SD), and median. All summaries will be by randomisation group. The primary analysis will be a linear regression of depression scores at 3 months including randomisation group and strata as factors and baseline depression scores as covariate. Potential confounders, apart from baseline and stratification variables, identified by clinical consensus, will be included in multivariable analyses when appropriate (e.g. when performing sub-group analyses). A mixed effects model will be used to analyse the longitudinal change in HADS scores and HeartQoL scores, adjusting for strata, incorporating random effects to model the homogeneity within cluster and the correlation within patient across time. Pre-planned subgroup analyses will be conducted for the primary outcome with respect to: *(i)* type of IHD (stable versus unstable); *(ii)* sex (female versus male); *(iii)* age (median split); *(iv)* depression status at baseline (< 10 versus ≥11; *≥11 is considered a moderate score*). A sensitivity analysis will be conducted. The statistical analysis will be transparent, subject to review and approval by the project team.

## Trial status

The first patient was included into the trial on 22 May 2020. Now (17 December 2020) we have 31 patients included into the trial of the 188 patients required.

## Discussion

The multi-disciplinary *eMindYourHeart* trial is highly relevant due to projections that IHD and depression will be among the top contributors to the disease-burden worldwide in 2020 [2, 11, 12]. CR programs show mixed efficacy with respect to targeting vulnerable patients who are at risk of refusing or dropping out of the program [17]. There is an absence of treatment for patients who screen positive for depression and anxiety, as screening for depression and anxiety in patients with IHD combined with treatment are far from universally implemented [20]. If proven effective, *eMindYourHeart* holds a strong potential for improving the quality of care of the increasing population of patients with IHD and comorbid depression and anxiety, with likely benefits to patients, families, the healthcare system and society at large due to potential reductions in direct and indirect costs. The trial evaluates both the efficacy and the cost-effectiveness of the intervention, which is adherent to the recommendations by the American Heart Association [43] and the European Society of Cardiology [44].

## Limitations

We are not able to blind patients to their condition, which may lead to bias and exaggeration of the effect [45]. The results may not be generalisable to the population of patients with IHD and comorbid depression and anxiety, as we cannot include patients who are not referred for CR or who refuse participation. Only patients who are able to use a computer and log into the platform are eligible. Some patients (e.g. depressed patients) may not have the motivation and energy to participate, which may lead to a potential selection bias.

## Conclusions

*eMindYourHeart* will provide new insights into the efficacy and acceptance of a therapist-assisted internet treatment for patients with IHD and comorbid depression and anxiety as part of standard CR that was designed with and for this patient group. This may increase the application’s quality and level of user acceptance [46, 47].

## Data Availability

Datasets from the study will be made available upon request to the corresponding author once the trial has been completed and all papers in the *eMindYourHeart* publication charter have been published.

## Abbreviations

AMI: Acute myocardial infarction
CONSORT: Consolidated standards of reporting trials
CR: Cardiac rehabilitation
DHRD: Danish cardiac rehabilitation database
DSMB: Data safety monitoring board
GDPR: General data protection regulation directive
GP: General practitioner
HADS: Hospital anxiety and depression scale
ICD: Implantable cardioverter defibrillator
IHD: Ischaemic heart disease
OPEN: Open patient data explorative network
RCT: Randomised controlled trial
REDCap: Research electronic data capture
SCAN: Schedules for clinical assessment in neuropsychiatry
SD: Standard deviation
TAU: Treatment as usual
QoL: Quality of life
